# Assessment of Peripheral and Central Auditory Processing after Treatment for Idiopathic Sudden Sensorineural Hearing Loss

**DOI:** 10.1055/s-0043-1776728

**Published:** 2024-03-15

**Authors:** Soheila Khakzand, Mohammad Maarefvand, Masoumeh Ruzbahani, Ardavan Tajdini

**Affiliations:** 1 Audiology Department, School of Rehabilitation, Iran University of Medical Sciences, Tehran, Iran; 2 Ear, Nose and Throat Department, Amir-Alam Hospital, Tehran University of Medical Sciences, Tehran, Iran

**Keywords:** sudden hearing loss, otoacoustic emission, evoked potentials, pitch perception, just-noticeable differences

## Abstract

**Introduction** When cases of idiopathic sudden sensorineural hearing loss (SSNHL) are treated successfully, most clinicians assume the normality and symmetry of the auditory processing. This assumption is based on the recovery of the detection ability on the part of the patients, but the auditory processing involves much more than detection alone. Since certain studies have suggested a possible involvement of the central auditory system during the acute phase of sudden hearing loss, the present study hypothesized that auditory processing would be asymmetric in people who have experienced sudden hearing loss.

**Objective** To assess the physiologic and electrophysiological conditions of the cochlea and central auditory system, as well as behavioral discrimination, of three primary aspects of sound (intensity, frequency, and time) in subjects with normal ears and ears treated successfully for SSNHL.

**Methods** The study included 19 SSNHL patients whose normal and treated ears were assessed for otoacoustic emissions, speech auditory brainstem response, intensity and pitch discrimination, and temporal resolution in a within-subject design.

**Results** The otoacoustic emissions were poorer in the treated ears compared to the normal ears. Ear- and sex-dependent differences were observed regarding otoacoustic emissions and pitch discrimination.

**Conclusion** The asymmetrical processing observed in the present study was not consistent with the hearing threshold values, which might suggest that the central auditory system would be affected regardless of the status of the peripheral hearing. Further experiments with larger samples, different recovery scenarios after treatment, and other assessments are required.

## Introduction

Sudden sensorineural hearing loss (SSNHL) is an emergency clinical otologic condition defined as the occurrence of 30 dB or more of hearing loss over 3 consecutive frequencies within 72 hours, 1 2 3 and its incidence is of 5 to 160 per every 100 thousand individuals. 3 4 This condition affects the quality of life of patients, for it limits their communication abilities; 4 it is unilateral in 95% of the cases, 4 5 without side preferences, 5 6 with equal incidence in both sexes and bilateral sequential involvement in less than of 2% of the patients. 6 7 8 It can damage both hearing and the vestibular system. 4 Aural fullness, 4 9 tinnitus, 3 9 and vertigo 9 are other common symptoms of SSNHL. Although there are some etiologies for SSNHL, no identifiable cause can be found in most cases, and these patients are classified as having “idiopathic” SSNHL. 7 9 The treatments for idiopathic SSNHL are controversial, 4 and the one most frequently used is oral steroid therapy. 2 However, if the treatment is administered within days or weeks of the onset of the SSNHL, 10 there is a high probability that it will be successful. Better prognosis for recovery has been reported for younger patients, and those with less severe hearing loss, lack of concomitant vertigo, and no delay between the onset of hearing loss and treatment. 3 7 11 It is worth mentioning that hearing may take three months to return to irs previous condition, before the onset of SSNHL. 12 13 14 The long-term follow-up studies 15 (> 5 years) on the hearing status of the affected ears showed no changes in hearing loss in most SSNHL patients. In certain subjects, hearing loss deteriorated over time and, in others, the hearing status improved, especially regarding the low frequency region, depending on alcohol consumption and vascular impairment. 15

The success of the treatment for idiopathic SSNHL is determined with hearing recovery, which is quantitatively expressed by the degree of improvement in pure tone average (PTA) after the administration of the treatment. 2 The more the hearing thresholds improve, the more successful the treatment. There are reports as well of hearing thresholds that remained asymmetric even after successful treatment and recovery. 16 17 However, the recovery of hearing thresholds following idiopathic SSNHL may be complete, partial, or it may not happen at all. 7 If the PTA returns to the normal range, most clinicians often assume the “normal auditory processing” of sound signals in the treated ear.

In fact, PTA only informs clinicians about the peripheral hearing system, 13 and idiopathic SSNHL may be due to abnormalities in the cochlea, auditory nerve, or central auditory system, or to a combination of peripheral and central auditory system involvement. 1 5 8 18 As a result, people with SSNHL may complain about their speech perception even after their audiometric thresholds have returned to the normal range and, in certain cases, speech perception scores in quiet or noise are not proportional to the degree of hearing loss and PTA, 9 19 20 even a long time after the treatment. 19 21 22 In addition, it has been shown that audiometric evaluation cannot inform about the hearing status in the ear affected by SSNHL. 15

The deterioration of speech perception after SSNHL cannot solely be justified by peripheral hearing status, since many factors such as central auditory processing and those pertaining cognition also play a roles in this regard. 18 Negative auditory plasticity after SSNHL in the central auditory system has been suggested as a cause of the deterioration of speech perception. 1 19 23 In SSNHL, auditory processing is often interrupted suddenly and unilaterally in contrast to other types of SNHL, in which alterations in auditory processing may occur over time and bilaterally, which may provide the hearing system time to use different strategies to cope with them. Moreover, imaging studies 1 have shown that SSNHL could change the function of the auditory cortex and certain non-auditory regions of the brain, even within the acute period of hearing loss. These reports may indicate the possibility of both peripheral and central auditory system involvement even after a successful treatment. 5 24 25

In addition to the speech perception problem, difficulty in locating sounds and vestibular issues have been reported in people with SSNHL. 26 27

Audiometry only measures the hearing threshold (that is, the detection of pure tones and speech sounds), while many aspects of auditory processing rely on the discrimination or identification of speech and non-speech sounds, which occur at suprathreshold levels. Speech perception, for example, requires the discrimination of different envelope patterns, tracking of pitch, and extraction of speech information from gaps and temporal cues, especially when background noise present. In addition to these acoustic cues, lexical familiarity and cognitive processes play a significant role in the perception of speech.

Despite the possibility of the central auditory system, only pure tone audiometry is being used for the assessment of the severity and recovery from SSNHL. To the best of our knowledge, the present study is the first that aimed not only to assess the aspects of sound involved in auditory processing (such as time, frequency, and intensity level) from the peripheral to the central systems in people experiencing SSNHL, but also at both threshold and suprathreshold levels. The hypothesis was that, in people treated successfully for SSNHL, auditory processing at the peripheral and central auditory systems would be asymmetrical. To test the hypothesis, the peripheral and central auditory systems were assessed objectively and behaviorally through conventional clinical audiologic tests at suprathreshold levels. The objective tests were conducted to provide insight about the physiologic status of the cochlea and brainstem. The behavioral tests assessed the discriminability of the auditory system in terms of intensity, frequency, and temporal aspects of sound perception. To remove the confounding effects of cognition and lexical factors, we used non-speech materials. Since suprathreshold auditory processing might be influenced by peripheral (cochlear) hearing loss, the present study only included subjects with unilateral idiopathic SSNHL whose hearing thresholds retuned to the normal range after the successful treatment, so that the results of suprathreshold auditory processing could not be influenced by peripheral hearing loss. Also, the normal ears of the participants were considered as control ears in a within-subject design, which enabled the control of the unknown individual factors related to unilateral idiopathic SSNHL.

## Methods

### Participants

Given the diversity in the pathophysiologic mechanisms of idiopathic SSNHL, all possible attempts were exhausted by setting strict rules for the patient selection process to make the sample more homogenous. The exclusion criteria were idiopathic SSNHL patients with fullness, vertigo or any vestibular or balance disorders (no vestibular system involvement), tinnitus, and poor speech perception in speech audiometry testing. All participants been treated successfully for SSNHL. They had normal PTAs and present otoacoustic emissions (OAEs), no middle ear problem or previous surgery (confirmed by type-A and present ipsilateral acoustic reflexes in the immittance testing), and intact auditory nerve (confirmed by the presence auditory brainstem responses, ABRs, with their latencies within the normal range). All of the subjects were referred from the same Ear, Nose and Throat Department, and they were treated with the same medical protocol and medications (75 mg of oral or intravenous prednisolone according to physician discretion, depending on the severity of the hearing loss tapered within the first two weeks of the onset of SSNHL), and were submitted to normal magnetic resonance imaging (MRI) scans. None of them had congenital or industrial hearing losses prior to SSNHL, and they were all younger than 60 years of age. Since sex and the laterality of the SSNHL could influence the results of the experiments, these two factors were also considered in the participant selection. Since full recovery of the auditory processing acuity might happen up to three months after the SSNHL treatment, the participants were included if the interval between the end of their treatment and the time of the experiments was of at least three months. Having satisfied these conditions, 19 (10 female) subjects with idiopathic SSNHL were included. In total, 4 male and 6 female patients had SSNHL in the left ear, and 5 male and 4 female participants had SSNHL in the right ear. Their mean age was of 28.18 ± 5.16 (range: 22 to 55) years, and 10 subjects presented sloping hearing loss, followed by 6 with rising and 3 with flat audiograms before the treatment and normal PTA (lower than 25 dB HL). All of the participants were tested in 2021 and 2022. The study design is within-subject and comparative. Before the administration of the test, all of the participants signed the consent form, and the study was approved by the institutional Ethics in Research Committee.

### Experiments

The audiometric evaluations were performed before and after the medical treatment from the frequencies of 0.25 kHz to 8 kHz in 5-dB steps, according to the Hughson *-* Westlake procedure: if the listener hears a presented sound, its intensity decreased by 10 dB, and if the listener does not hear a presented sound, its intensity increased by 5 dB. At each frequency, the hearing threshold is the lowest intensity at which the patient hears the tone at least 50% of the time. 28 The audiometry and the subsequent behavioral tests were performed using the AC40 clinical audiometer (Interacoustics A/S, Middelfart, Denmark) unilaterally, with the circumaural TDH-39 audiometric earphones (Telephonics, Farmingdale, NY, United States). In addition, immittance measurements were performed for all participants.

#### Electrophysiological Tests

Electrophysiologic tests were used to assess the cochlea and auditory nerve after treatment. Through the OAEs, we evaluated the presynaptic function of the cochlea, especially the function of the outer hair cells (OHCs). The presence of OAEs indicates the integrity of the conductive path for sound to the cochlea and of the function of the OHCs. Transient evoked OAEs (TEOAEs), and distortion product OAEs (DPOAEs), were measured in normal and treated ears separately. The OAEs test was performed using the Madsen Capella device (GN Otometrics, Taastrup, Denmark).

The ABRs can be used to assess the integrity of the postsynaptic function of the auditory nerve and low brainstem centers for sound processing with either non-speech (click ABR and tone-burst ABR), or speech stimuli (speech ABR or sABR). While click and tone-burst ABRs reveal the simple synchronization of the neural depolarization along the auditory pathway, sABRs are more informative regarding the processing of spectral and temporal aspects of complex stimuli (such as speech) in the brainstem and higher centers of the central auditory system. 29 Moreover, we preferred the sABR to the click-ABR in the present study because it is less affected by peripheral hearing losses, so it could show the function of the central auditory system. The sABRs were measured in the normal and treated ears separately using the Bio-logic device (Natus Medical Incorporated, Middleton, WI, United States), and the ER-100 (MAICO Diagnostics, Berlin, Germany) insert earphones.

***TEOAEs.*** The TEOAEs were recorded following the presentation of clicks (transient stimuli) in the intensity of 80 dB peak sound pressure level (SPL). These emissions were considered present if their amplitudes were 6 dB higher than those of the noise levels (that is, a signal-to-noise ratio [SNR] of 6 dB) and if the correlation involving OAEs for different clicks reached at least 80%. The amplitudes of TEOAEs at frequencies of 1 kHz, 2 kHz, and 4 kHz were analyzed and compared between the treated and normal ears.

***DPOAEs.*** The DPOAEs are the distortion products of activities in the cochlea in response to a pair of tones presented simultaneously. In the present study, the frequency of the second tone (called f2) was 1.22 times that of the first tone (f1), and f1 and f2 had the intensity levels of 65 dB SPL and 55 dB SPL respectively. Moreover, DPOAEs were recorded at a frequency equal to 2 x f1 - f2, which presented the largest amplitude in the frequency range of 1 kHz to 4 kHz, and were considered present if their amplitudes were of 6 dB SNR. The amplitudes of DPOAEs in the treated and normal ears were analyzed and compared in octave frequencies ranging from 0.5 kHz to 4 kHz.

***sABR.*** The sABR was used to reveal the difference in the processing of a consonant-vowel (CV) speech stimuli in the normal and treated ears; the stimuli used were /da/. The duration of the stimulus was of 40 ms, and the rate of stimulation was of 10.9 stimuli per second. The total number of stimuli presented to each ear was of 6 thousand. The positive electrode (or non-inverting electrode) was placed on the vertex, and the negative electrode (inverting electrode), on the right earlobe (to test the right ear) and left ear (to test the left ear). The ground electrode was placed on the forehead. The polarity of the stimulation was alternate. The normal and treated ears were evaluated separately and monaurally.

The sABR waveforms consist of several components named as V, A, C, D, E, F, and O. The V and A components reveal the response of the brainstem to the onset of the consonant in a CV stimulus, while other components are likely to be related to the transition from consonant to vowel and the onset of vowel (component C) and the offset of the stimulus (component O). The harmonic structure of the vowel gives rise to the frequency-following response (FFR, components D, E, and F). 29 Since components V and A are more stable in sABR, their latencies in the normal and treated ears were recorded and compared.

#### Behavioral Tests

To measure the just-noticeable difference (JND) in three aspects of sound perception – pitch and loudness discrimination and temporal resolution –, we used the following behavioral tests respectively: the difference limen for frequency (DLF), the difference limen for intensity (DLI), and the gap-in-noise (GIN) tests, which were performed after they were described in plain language followed by a training session. The tests were administered in an acoustic booth, and their items were presented at the level that was most comfortable to the participants. The order of the tested ears was randomized.

***DLF*** . The DLF measured the JNDs for frequency (different pitch perception) in octave frequencies from 0.5 kHz to 4 kHz. At each frequency, tones were modulated from 0 Hz (no modulation) to 5 Hz. The participants were asked to listen to the pitch of a base frequency and then raised their hands whenever they perceived a modulation in pitch. The thresholds or JNDs were searched in a descending-ascending manner. The minimum number of changes in frequency, which induced a modulation in pitch in two out of three runs, was recorded as the JND for a frequency.

***DLI.*** The DLI assessed the JNDs for intensity (different loudness perception) in octave frequencies from 0.5 kHz to 4 kHz. The participants were asked to listen to the loudness of a base frequency and then raised their hands whenever they perceived a modulation in loudness for that frequency. The two tones could differ in intensity. from as low as 0 dB (no modulation in intensity) to as high as 5 dB. The thresholds or JNDs were searched in a descending-ascending manner. The minimum number of changes in intensity, which induced a modulation in loudness in two out of three runs, was recorded as the JND for intensity.

***GIN.*** The GIN measured the minimum perceived gap between sequences of acoustical events that enabled people to perceive them separately. This minimum gap indicated the temporal acuity of the hearing system or threshold for temporal resolution. There were 10 different gaps of different lengths (2, 3, 4, 5, 6, 8, 10, 12 , 15 and 20 ms), and each was presented 6 times, yielding 60 gaps, which were randomly distributed in 30 white noise segments with a length of 6 s. There could be between 0 to 3 gaps in each segment. The GIN threshold was defined as the minimum gap (in ms) which a participant could detect correctly in 4 out of 6 repetitions.

***Statistical analysis.*** The statistical analysis was performed using the SPSS Statistics for Windows (SPSS Inc., Chicago, IL, United States) software, version 17.0. The data for different tests either followed normal distribution or distribution with similar standard deviations (SDs) and sample sizes, which made the statistical analysis robust in the case of a small departure from normality observed in certain analyses. In the descriptive analysis, we have summarized the average and SD values for each measurement.

In the statistical analysis, the score on each test (dependent variables) was compared in terms of “condition” (that is, normal and treated ears as a within-subject factor), while the “laterality of SSNHL” (that is, left or right treated ear) and “sex” variables were included in the model (as between-subject factors). Since, the PTA may not be representative of all frequencies and the scores in the aforementioned tests might be influenced by hearing thresholds at measuring frequencies, we decided to enter a “hearing threshold” variable as a covariate to account for the effect of audibility at each frequency measured. Therefore, we used analysis of covariance (ANCOVA) to answer the hypothesis. The significance level (α) was set at 0.05. To control for type-І error in post-hoc testing, the Bonferroni correction coefficient was applied prior to the analyses. The Pearson correlation was used in cases in which the relationships regarding different test scores had to be assessed. For the comparison of the PTAs of the normal and treated ears, the paired *t* -test was used.

## Results

The average and SD of the PTA in the normal ear was of 13.4 ± 6.1 (range: 6.6 to 31.6 dB HL); in the treated ear, these values were of 47.1 ± 12.51 (range: 30 to 71.6 dB HL) and 16.2 ± 5.2 (range: 8.3 to 28.3 dB HL) before and after treatment respectively. Despite the fact that the PTA values in the treated ears returned to the normal range, there was a significant difference between PTA values in the treated and normal ears after treatment (t = 2.57; *p* = 0.019). The hearing thresholds throughout the frequency range in the treated ear before and after treatment are shown in Table 1 .

**Table 1:** Hearing thresholds in the treated ear before and after treatment

				Before treatment						After treatment			
				Frequency (kHz)						Frequency (kHz)			
Treated ear	Sex	0.25	0.5	1	2	4	8	0.25	0.5	1	2	4	8
Right	Female	50	45	30	20	20	30	20	15	20	20	15	20
Right	Male	50	60	70	65	75	65	20	25	20	25	20	30
Left	Female	30	40	50	40	40	40	30	15	15	10	15	20
Left	Female	45	45	50	60	70	75	15	20	25	25	20	30
Left	Male	60	50	45	30	25	20	30	15	15	15	15	10
Right	Male	45	60	70	65	65	50	25	15	25	20	25	25
Left	Female	50	40	45	60	70	80	20	15	20	15	25	45
Left	Female	30	40	50	35	30	25	15	10	15	15	10	25
Right	Male	60	50	50	70	80	90	30	20	25	25	15	50
Left	Male	45	55	60	70	75	80	20	20	20	25	20	45
Left	Female	20	30	30	30	35	45	15	15	15	15	15	30
Right	Female	55	50	55	40	30	25	20	15	10	15	10	10
Right	Female	60	60	40	20	10	10	15	15	15	10	10	10
Right	Male	50	40	35	20	25	20	20	10	15	10	15	20
Left	Male	30	30	40	50	60	90	25	20	20	20	25	50
Left	Female	60	60	50	40	40	25	15	10	10	15	10	15
Right	Male	50	40	30	35	20	20	15	15	10	10	10	20
Left	Male	50	50	40	30	35	45	20	15	15	15	20	25
Right	Female	40	50	60	70	60	70	15	15	15	15	20	40

### Electrophysiological Tests

The descriptive analysis of the electrophysiological tests is shown in Table 2 (TEOAE, DPOAE) and Table 3 (sABR).

**Table 2:** Average and SD values of TEOAE and DPOAE amplitudes

	TEOAE (in dB SPL)		DPOAE (in dB SPL)	
	Male	Female	Male	Female
Normal ear	3.5 ± 8.5	-1.2 ± 9	8.6 ± 7.2	7.6 ± 11
Treated ear	1.2 ± 8.5	-3.2 ± 9.3	8.4 ± 10	5 ± 9

Abbreviations: DPOAE, distortion product otoacoustic emission; SD, standard deviation; SPL, sound pressure level; TEOAE, transient evoked otoacoustic emission.

**Table 3:** Average and SD values of the latencies of the V and A components of the sABR

	V component (ms)		A component (ms)	
	Male	Female	Male	Female
Normal ear	6.5 ± 0.3	6.5 ± 0.1	7.3 ± 0.3	7.3 ± 0.3
Treated ear	6.6 ± 0.2	6.5 ± 0.1	7.3 ± 0.1	7.3 ± 0.1

Abbreviations: sABR, speech auditory brainstem response; SD, standard deviation.

#### TEOAEs and DPOAEs

**Fig. 1:**
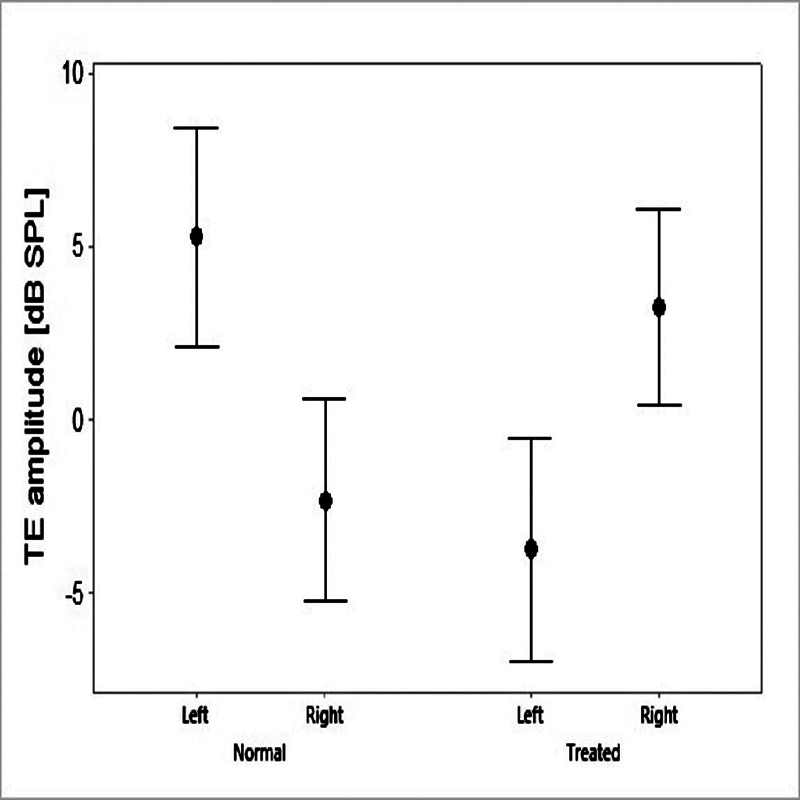
Mean and 95% confidence interval values for the comparison of TEOAE amplitudes in the normal and treated ears of male and female patients.

There was a significant difference regarding conditions in interaction with the laterality of the SSNHL (f = 21.23; df = 1; *p* < 0.005) after the correction by the covariate factor. The left normal ears presented significantly higher TEOAE amplitudes than the right normal ears, and the opposite was true for the treated ears ( Figure 1 ).

**Fig. 2:**
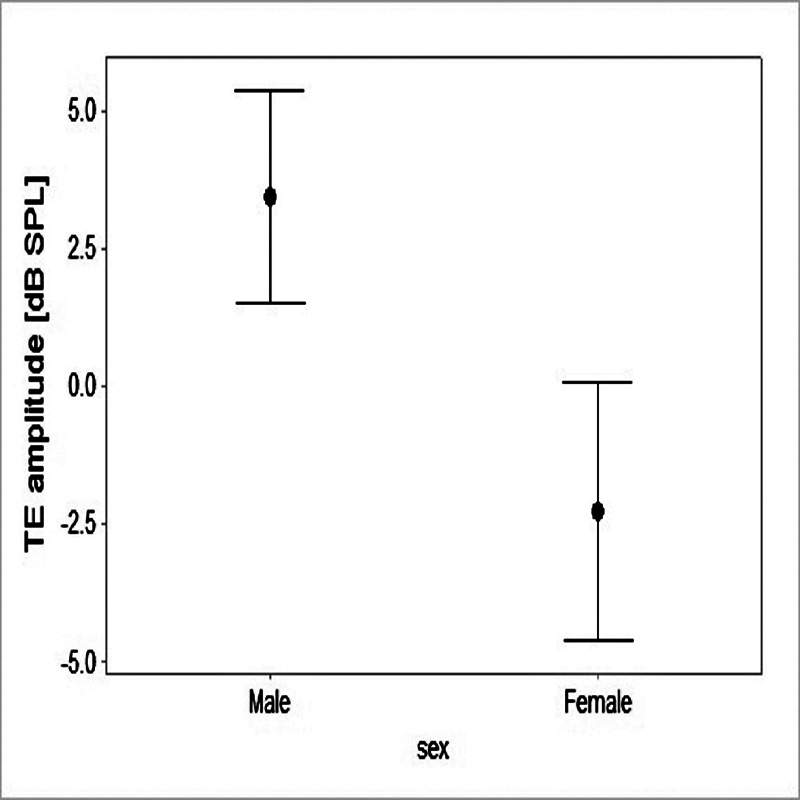
Mean and 95% confidence interval values of TEOAE amplitudes in male and female patients.

**Fig. 3:**
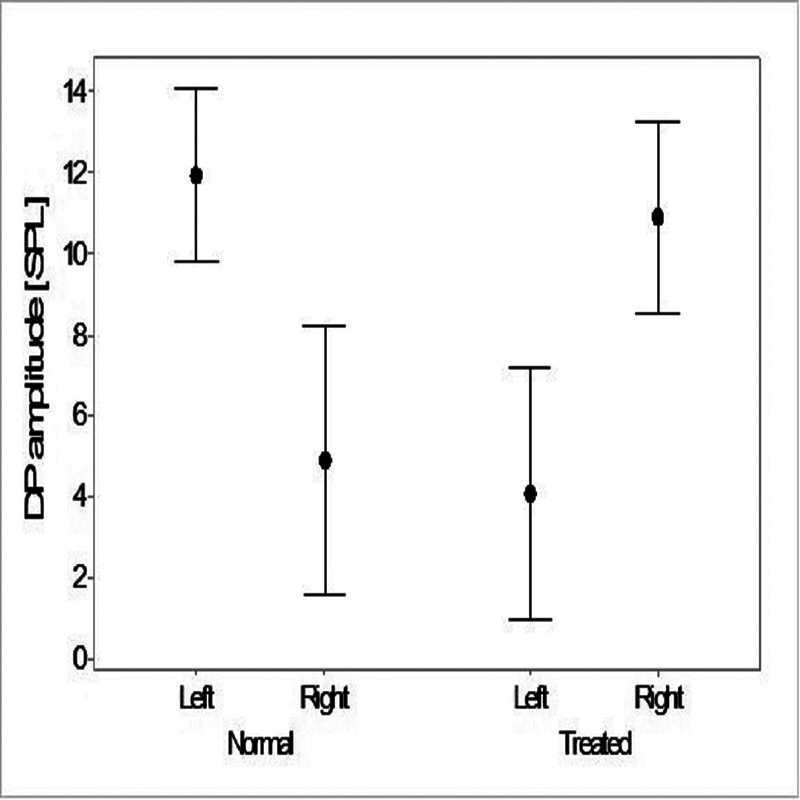
Mean and 95% confidence interval values for the comparison of DPOAE amplitudes in the normal and treated ears of male and female patients.

Moreover, the effect of sex was significant (f = 11.33; df = 1; *p* = 0.001), and the male participants presented significantly higher TEOAE amplitudes ( Figure 2 ). No significant correlation was found regarding hearing threshold values and TEOAE amplitudes in the same frequency range of 1 kHz to 4 kHz. There was also a significant difference regarding conditions in interaction with the laterality of the SSNHL (f = 21.26; df = 1; *p* < 0.005) after the correction by the covariate factor in the DPOAEs test ( Figure 3 ). There was no significant correlation involving hearing threshold values and the DPOAE amplitudes in the same frequency range of 0.5 kHz to 4 kHz.

#### sABR

**Fig. 4:**
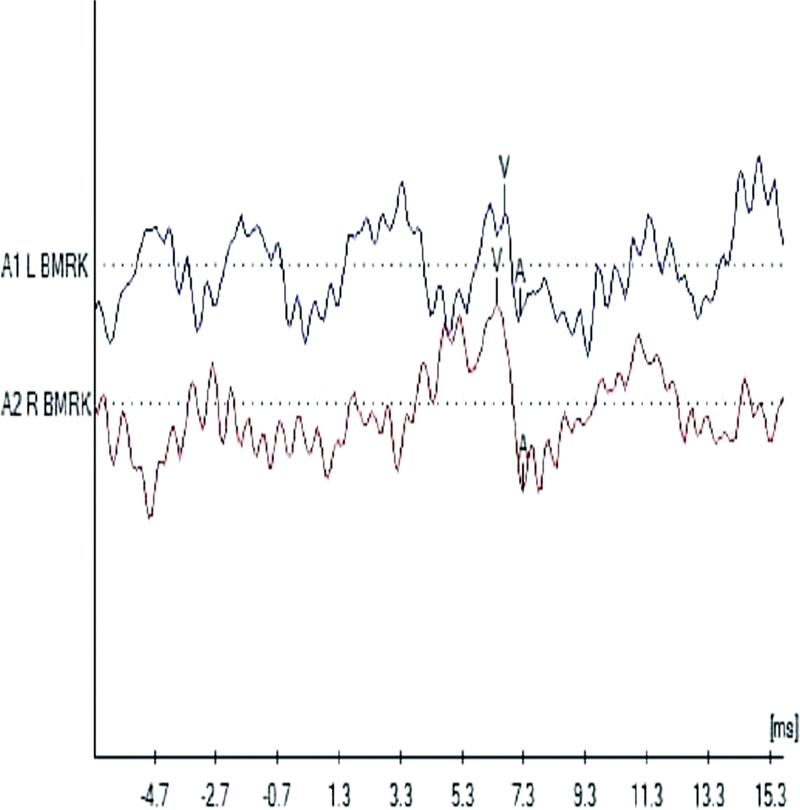
Examples of sABR traces in the normal (upper trace) and treated ears (lower trace).

An example of sABR trace measured is shown in Figure 4 . This difference was not significant difference between different conditions. The correlation regarding the values for latency of the V or A components and the average of the hearing threshold values was not significant.

### Behavioral Tests

#### DLF and DLI

**Fig. 5:**
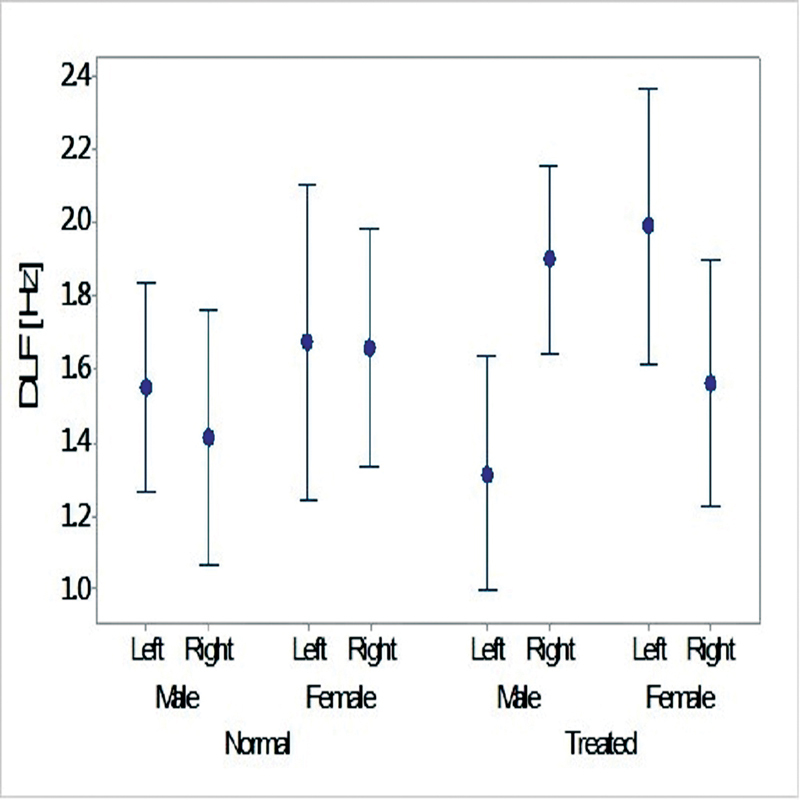
Mean and 95% confidence interval values for the effects of sex and laterality of the SSNHL on the DLF results regarding the normal and treated ears.

Table 4 shows the average and SD values for the DLI, DLF and GIN tests in the normal and treated ears. While there was no significant difference involving conditions in the DLI test, a significant interaction was observed between the laterality and sex in the DLF test in the two ears after the correction by the covariate (f = 5.93.; df = 1; *p* = 0.016) ( Figure 5 ) shows . There was no significant correlation regarding the DLI test and hearing threshold values, nor regarding hearing thresholds and the DLF test in the same frequency range of 1 kHz to 4 kHz.

**Table 4:** Average and SD values of the DLI, DLF and GIN tests

		DLI (dB)		DLF (Hz)		GIN (ms)	
Ear	Sex	Average	SD	average	SD	Average	SD
Normal ear	Male	1.4	± 0.6	1.6	± 0.7	3.94	± 0.3
	Female	1.3	± 1.6	1.7	± 0.8	3.7	± 0.2
Treated ear	Male	1.5	± 0.7	1.7	± 0.6	4.7	± 0.8
	Female	1.1	± 0.1	1.8	± 0.8	4	± 0.7

Abbreviations: DLF, difference limen for frequency test; DLI, difference limen for intensity test; GIN, gap-in-noise test; SD, standard deviation.

#### GIN

There was no significant difference between the normal and treated ears in the form of main or interaction term between variables. Neither was there a significant relationship involving the average of the hearing thresholds and the GIN threshold values.

## Discussion

The present study assessed the function of the peripheral and central auditory systems using behavioral, physiological, and electrophysiological tests to challenge the assumption of normality and symmetry of sound processing after the normalization of PTAs in SSNHL patients. It also provided an opportunity to examine the auditory system without the presence of audibility issues in patients who only experienced SNHL for a short period.

In terms of the assessment of the peripheral sound processing, the PTA returned to the normal range, and there were normal OAEs in the treated ears after the successful treatment, which is in line with the reports of previous studies. 12 30 The return of the PTA and OAEs to their normal range confirmed the functionality of the cochlea behaviorally and physiologically respectively. However, there was an asymmetry even in audibility (PTAs) regarding the detection of sounds, which is also supported by previous findings. 7 16 Moreover, different patterns of TEOAEs and DPOAEs have been observed in the patients treated for SSNHL compared with those with normal hearing. While many studies 31 32 have shown higher TEOAE and DPOAE amplitudes (larger OAEs) in the right ears of normal hearing subjects, no superiority or advantage in OAE production has been shown regarding the right or left ears of SSNHL patients. This advantage ( larger OAEs in the right ears) is considered an initial factor for the development of cerebral laterality and specialization of the left hemisphere for speech processing. 31 As Figures 1 and 2 show, in the present study, no significant advantage was found in terms of the amplitudes of OAEs in patients with left treated ear and right normal ear and vice versa. This might not be related to different audibility in the two ears, since the possible effect of the hearing threshold was adjusted with inclusion of a covariate in the analysis, and there was no significant correlation between hearing thresholds and OAE amplitudes. In addition, the participants did not have cochlear hearing loss after the treatment, and they were tested after their hearing system had had enough time to recover. This may indicate that not all OHCs return to their normal function, even three months after the treatment, which could be due to a lack of complete regulation of the blood flow to the cochlea. 33

The advantage of the right or left ear has also been attributed to the central auditory system. 34 Although OAEs reflect presynaptic activities of the cochlea and their amplitudes indicate the output of these activities, their amplitudes are also regulated by the centrifugal innervation from medial olivary complex down to the cochlea. 32 Previous research 35 has shown that the function of the centrifugal innervation is stronger in the right ear of subjects with normal hearing and this is highly correlated with speech perception in noise. According to Noguchiet al., 19 this may due to the negative plasticity in the central auditory system after SSNHL, and it may take time for speech perception scores to return to optimal values. 9 Therefore, the absence of the larger OAEs in the right ear may indicate changes in the central auditory system even after successful treatment in SSNHL patients.

Researchers have suggested an interruption in the blood supplied by the anterior inferior cerebellar artery to the vestibular and cochlear systems as a theoretical mechanism for SSNHL. Interruptions in the blood supply may impair cochlear structures through deficiency in oxygen delivery to the cochlea, but the impairment may extend to the supporting cells as well. Therefore, the supporting cells may not be able to take up glutamate from the synaptic cleft, and the accumulation of glutamate in the perilymph may impair the dendrites of the auditory neurons. 36 37 The anterior inferior cerebellar artery also supplies part of the brainstem. 38 39 In addition, imaging studies 40 have reported an acute inflammatory process in the cochlea and cerebral vessels.

Most of the previous studies 31 32 have reported that female subjects with normal hearing present higher TEOAE amplitudes. It is believed that the sexual differentiation of the brain (during weeks 8 to 24 of gestation) overlaps the development of the auditory system. The exposure of the male fetus to high levels of testosterone in this period may cause a decrease in the production of OAEs through worsening of the function of the cochlear amplifiers. However, lower TEOAE amplitudes in female subjects were observed in the present study, which may indicate that full recovery of TEOAEs after SSNHL may take time, or it may indicate changes in the central auditory system, as asymmetrical OAE amplitudes are attributed to different exposure to sexual hormones or to effects of the efferent auditory neurons originating from the brainstem.

The presence of ABR with normal latencies indicated normal detection of sounds by the brainstem. In the present study, no differences were found regarding the latencies of components V or A of the treated and normal ears. The latencies of components V or A in sABR are related to the onset of the consonant in CV stimuli. 25 Neither were there significant differences between the treated and normal ears in terms of he GIN and DLI results, which were discrimination tasks at suprathreshold levels. In sABR, the auditory neurons are excited synchronously to the onset or rise time of sounds' envelopes. Moreover, in the GIN test, the subjects track changes in the envelope of wide-band noise over time, and in the DLI test, they discriminate changes in modulation of the envelope of a reference sound. It seems that restoring audibility after successful treatment for SSNHL may enable people to follow or discriminate envelope or intensity changes normally and symmetrically between treated and normal ears. In contrast to the DLI and GIN results, there was a significant difference between the treated and normal ears in the DLF experiment (that is, pitch discrimination). It may be reasonable to think that restoring audibility might not be enough for symmetrical pitch processing. The asymmetrical processing varied between genders only in the treated ears. Figure 5 shows the changes in pitch discrimination in SSNHL. The present study showed that, in general, male subjects had more lateralized pitch perception than female subjects when the perception of pitch required memory. While previous studies have proposed that the right auditory cortex and hemisphere are more active in pitch discrimination, this is the left hemisphere that shows more activation in the planum temporale region, especially in male patients in the pitch tasks in which memory plays a role. 42 Since the method to assess pitch discrimination required the participants to compare modulations in pitch with the memory of a base tone, the poorer performance of the right ear might be understandable, as inputs the from right ear cross to the left hemisphere and vice versa. Therefore, the impairment to the right ear might a have more negative effect on pitch discrimination. This pattern was more pronounced in the male subjects of the present study than in the female patients ( Figure 5 ). 42

Finally, we should emphasized that the present study was performed with very strict criteria in terms of participant selection to control for the heterogeneous nature of idiopathic SSNHL. Asymmetrical physiological activity of the cochlea and pitch discrimination were observed in patients who did not present hearing loss after the treatment, and all comparisons were performed after removing differences in hearing thresholds between ears (covariate factor). All the tests were performed at suprathreshold levels, and none of the participants complained about speech perception, vertigo, fullness, or tinnitus after the treatment. Although the values for both ears in different experiments were within the range reported for listeners with normal hearing, 43 the participants of the present study do not represent the general population with SSNHL. If either audibility recovery is suboptimal or there are other concomitant problems, more asymmetrical processing in the abnormal range and complaints regarding speech understanding may be expected from SSNHL patients.

It is worth mentioning that the effect of sex on pitch was only observed in the treated ears of the small number of patients included in the present study. The assessment of the interaction between gender and pitch perception or the effects of gender on OAEs after SSNHL require experimental methods designed to control for gender-related factors, different experimental tasks, and large samples of subjects with normal hearing, hearing impairment, and a wide range of SSNHL patients (including those who have experienced full recovery, as well as those whose hearing did not improve).

## Conclusion

Changes in the peripheral and central auditory systems were observed even after successful treatment for SSNHL. Despite the strict inclusion criteria adopted in the present study, it is necessary to replicate it with larger samples and different rates of successful treatment before generalizing its findings to the SSNHL patients.
